# Minimally Invasive Treatment of a Complex Tibial Plateau Fracture with Diaphyseal Extension in a Patient with Uncontrolled Diabetes Mellitus: A Case Report and Review of Literature

**DOI:** 10.7759/cureus.599

**Published:** 2016-05-04

**Authors:** Ashok K Rathod, Rakesh P Dhake, Aditya Pawaskar

**Affiliations:** 1 Department of Orthopaedics, LTMMC & LTMGH, Sion, Mumbai

**Keywords:** tibial plateau fractures, schatzker, k wires

## Abstract

Fractures of the proximal tibia comprise a huge spectrum of injuries with different fracture configurations. The combination of tibia plateau fracture with diaphyseal extension is a rare injury with sparse literature being available on treatment of the same. Various treatment modalities can be adopted with the aim of achieving a well-aligned, congruous, stable joint, which allows early motion and function.

We report a case of a 40-year-old male who sustained a Schatzker type VI fracture of left tibial plateau with diaphyseal extension. On further investigations, the patient was diagnosed to have diabetes mellitus with grossly deranged blood sugar levels. The depressed tibial condyle was manipulated to lift its articular surface using K-wire as a joystick and stabilized with an additional K-wire. Distal tibial skeletal traction was maintained for three weeks followed by an above knee cast. At eight months of follow-up, X-rays revealed a well-consolidated fracture site, and the patient had attained a reasonably good range of motion with only terminal restriction of squatting.

Tibial plateau fractures with diaphyseal extension in a patient with uncontrolled diabetes mellitus is certainly a challenging entity. After an extended search of literature, we could not find any reports highlighting a similar method of treatment for complex tibial plateau injuries in a patient with uncontrolled diabetes mellitus.

## Introduction

Since the tibial plateau is a major weight-bearing portion of the lower extremity, fractures involving it affect the function and stability of the limb. Schatzker types IV, V, and VI are considered as complex tibial plateau fractures, often associated with soft tissue injury, a high risk of wound complications, and difficult reduction [1]. In the current medical literature optimal treatment of these fractures remains controversial and challenging with no consensus available about the best approach to treat them. Among the different options available, the two surgical methods most commonly in use are plating and hybrid external fixation [1]. Open reduction and internal fixation (ORIF) allows anatomic reduction and stable fixation, but extensive exposure is often required. The combination of original injury damage with the extensive surgical approach, led to high rate of complications including wound healing problems and infection [2-3]. Attempts have been made in the past to reduce the incidence of complications by using less extensive exposure and indirect means of reduction. External fixation requires minimal surgical exposure but causes inconvenience and requires careful maintenance, along with the possibility of pin tract infections and subsequent collapse of the fracture fragments [4]. The management of such patients becomes more challenging when they have associated comorbidities like diabetes mellitus. Complications related to bone and soft-tissue healing, and infection associated with diabetes mellitus have been well documented in the past [5-6].

We therefore consider worthwhile discussing a middle path of minimally invasive method for treating complex tibial plateau fracture (type VI Schatzker) with diaphyseal extension in a patient with uncontrolled diabetes mellitus. Informed consent was obtained from the patient for this study.

## Case presentation

**Figure 1:**
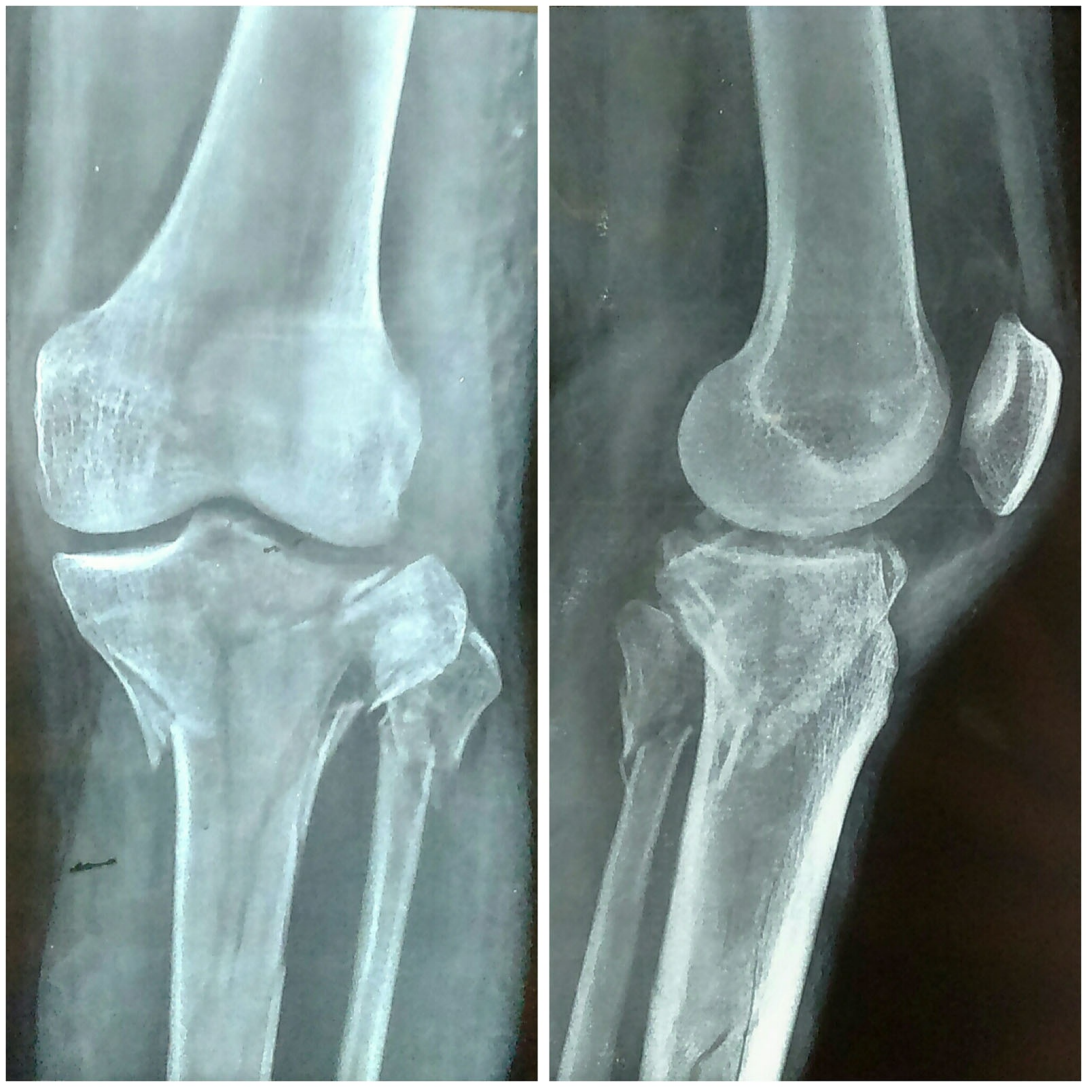
Anteroposterior and lateral X-ray views following injury.

A 40-year-old male, presented to our emergency department with closed injury to the left knee following a road traffic accident. On examination, he had a swollen, tender joint and restricted motion of the left knee without any neurovascular deficit or signs of compartment syndrome. The limb was initially splinted using a plaster of Paris (POP) back splint and subjected to radiological evaluation. Anteroposterior and lateral knee view radiographs revealed a complex comminuted fracture of the left tibial plateau with metaphyseal and diaphyseal extension (Figure 1).

**Figure 2:**
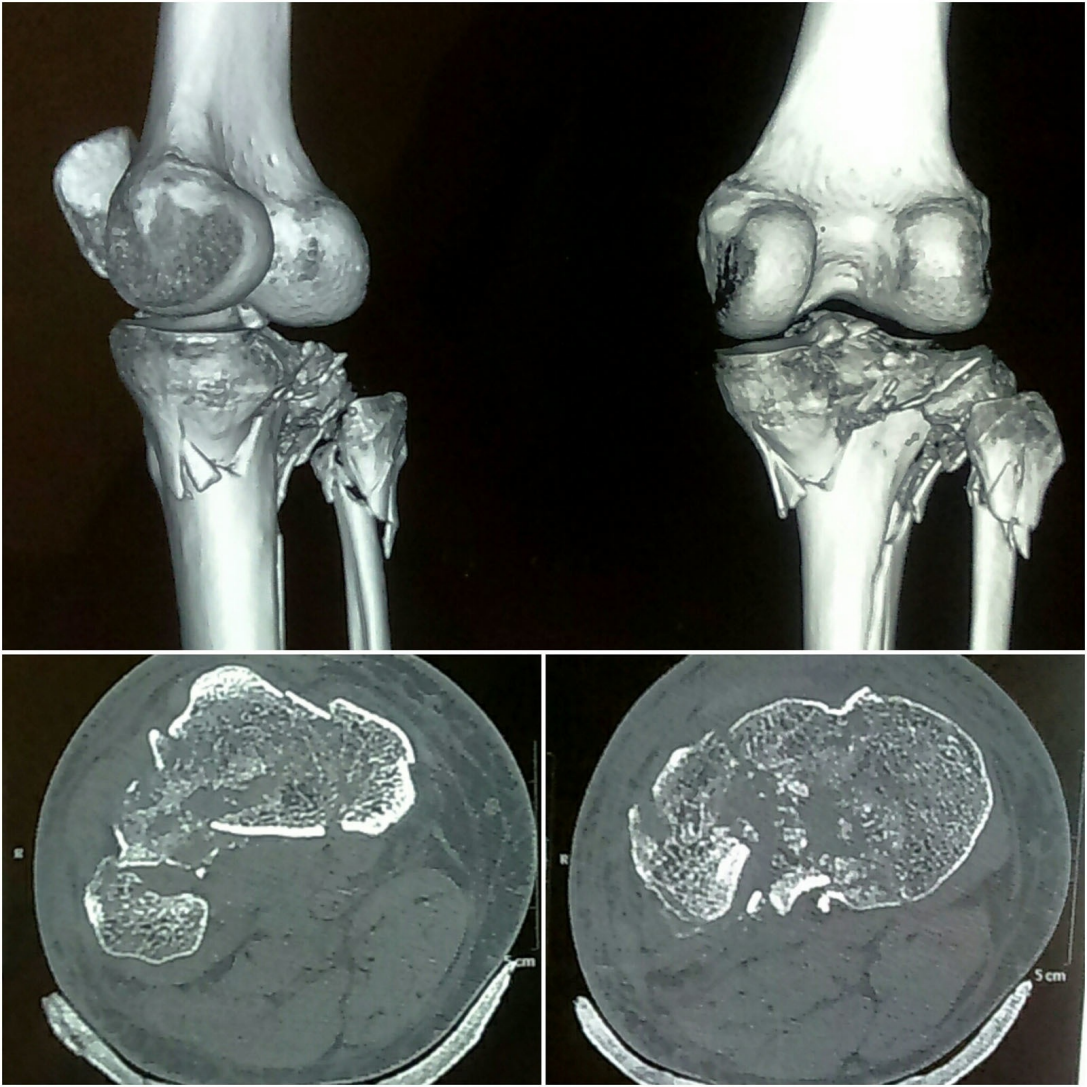
CT scan images depicting the fracture morphology.

A knee computed tomography (CT) scan was obtained for accurate fracture evaluation and was classified as Schatzker type VI according to Schatzker et al. [7] (Figure 2).

On further investigations, he was diagnosed to have diabetes mellitus with grossly deranged blood sugars. Though the medial and central articular surface appeared to be well maintained, the lateral articular surface was depressed and rotated downwards.

**Figure 3:**
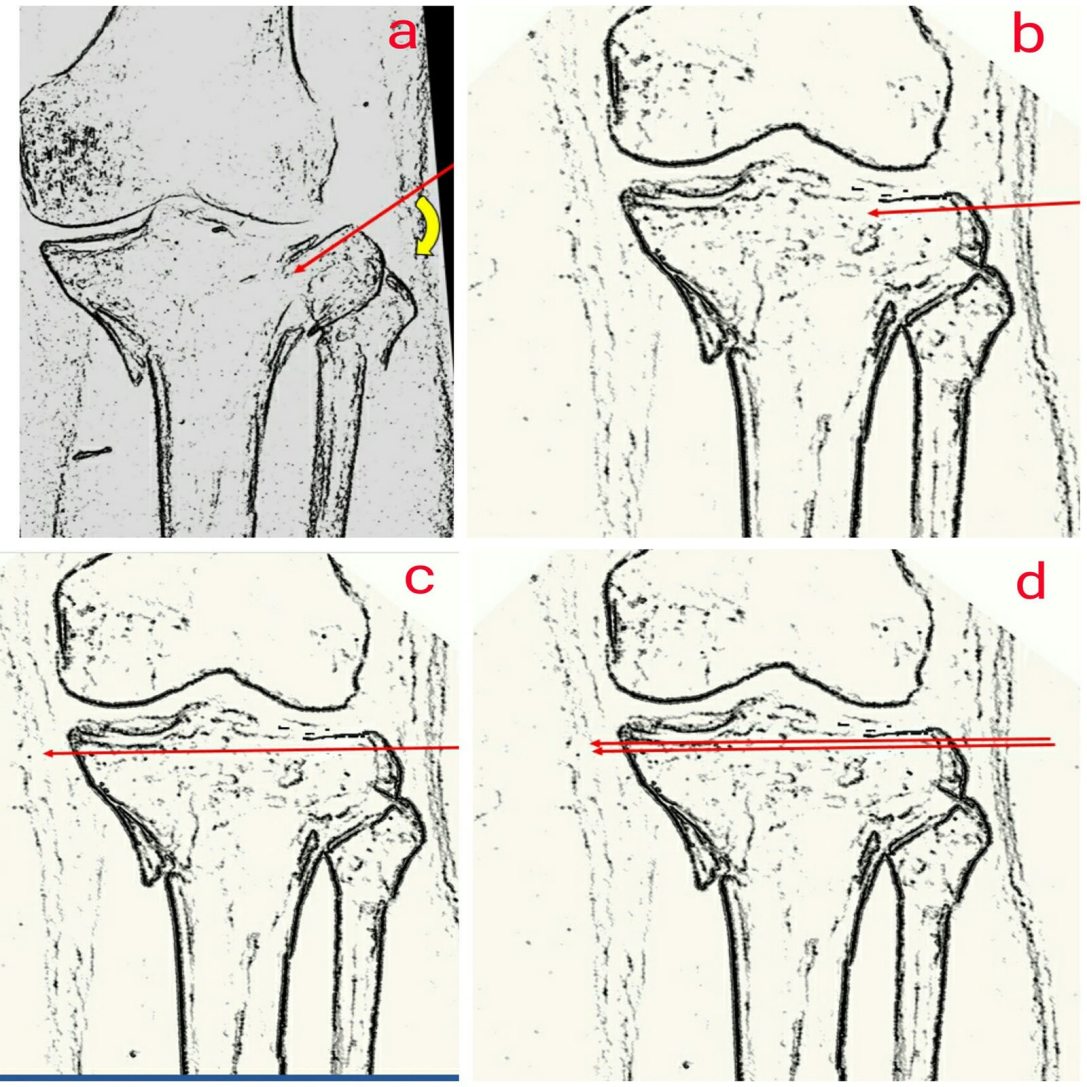
Diagrammatic illustration of the technique involving manipulation of fracture fragments using K-wire as a joystick.

A 6.5 mm Steinmann pin was inserted in distal tibia under local anesthesia and a heavy traction force of 10% of the body weight was applied. A traction radiograph defined the fracture morphology more clearly but the depressed rotated lateral articular surface could not be corrected on traction. Considering the complications of ORIF in a patient with uncontrolled sugar levels, it was decided to treat the patient with a minimally invasive technique that would elevate the lateral articular surface causing least damage to the surrounding soft tissues. Five days after the injury, the patient was taken for surgery under regional anesthesia. During the operation, the patient was positioned supine and the part was cleaned and draped in such a manner that the limb was free for manipulation during the operation. A thick K-wire was passed under the articular surface of fractured lateral condyle just short of the fracture site (Figure 3A).

**Figure 4:**
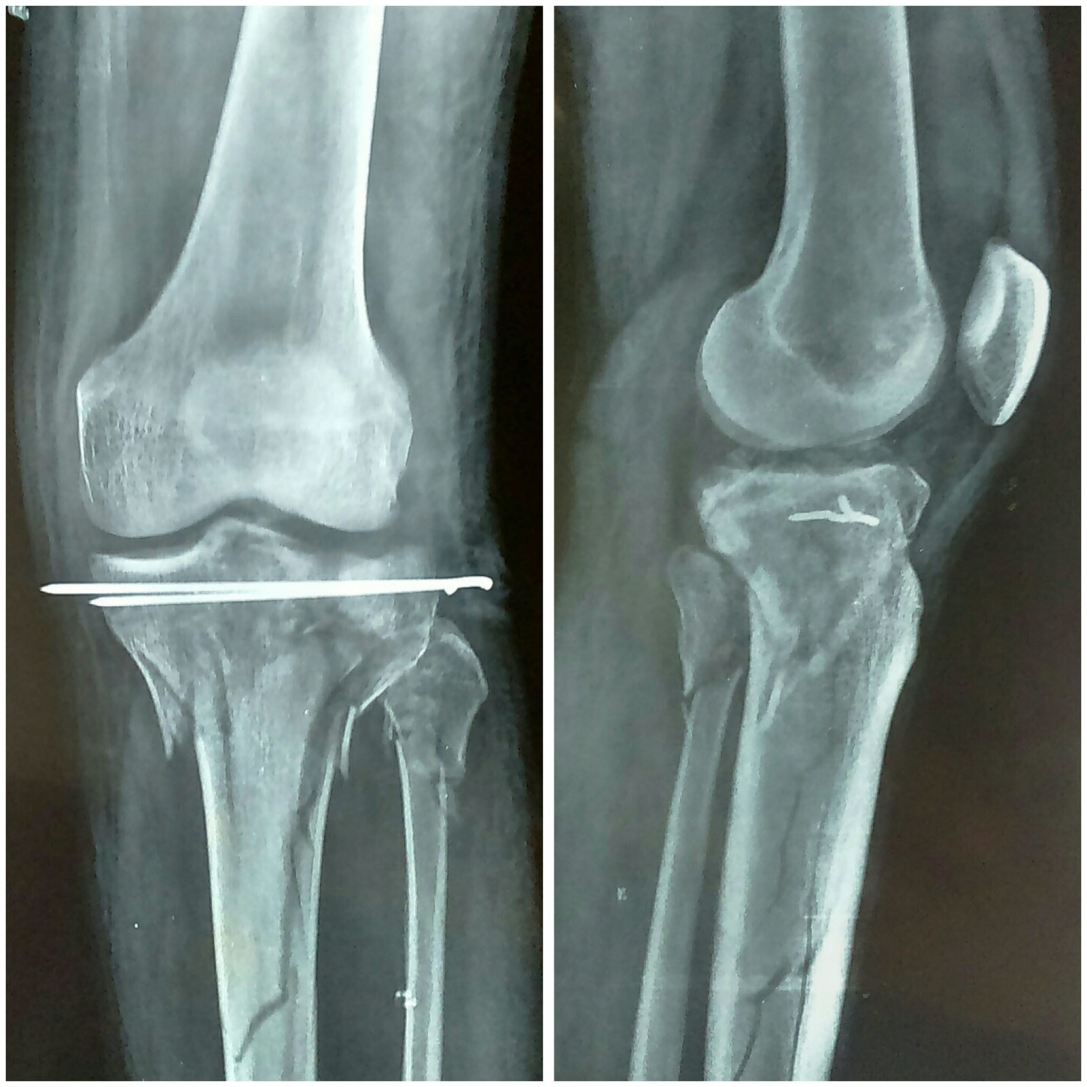
Immediate postoperative X-ray views: anteroposterior and lateral.

Using the K-wire as a joystick, the fractured lateral condyle was manipulated so as to lift its depressed articular surface, which was confirmed under constant fluoroscopic guidance (Figure 3B). A reduction clamp was used to bring together the fracture fragments. After confirming the reduction under an image intensifier, the K-wire was advanced parallel to the joint to engage the opposite cortex (Figure 3C). Another K-wire was passed parallel to the first wire under the articular surface to increase the stability (Figure 3D). Elevation of the articular surface was confirmed on immediate postoperative X-rays (Figure 4).

**Figure 5:**
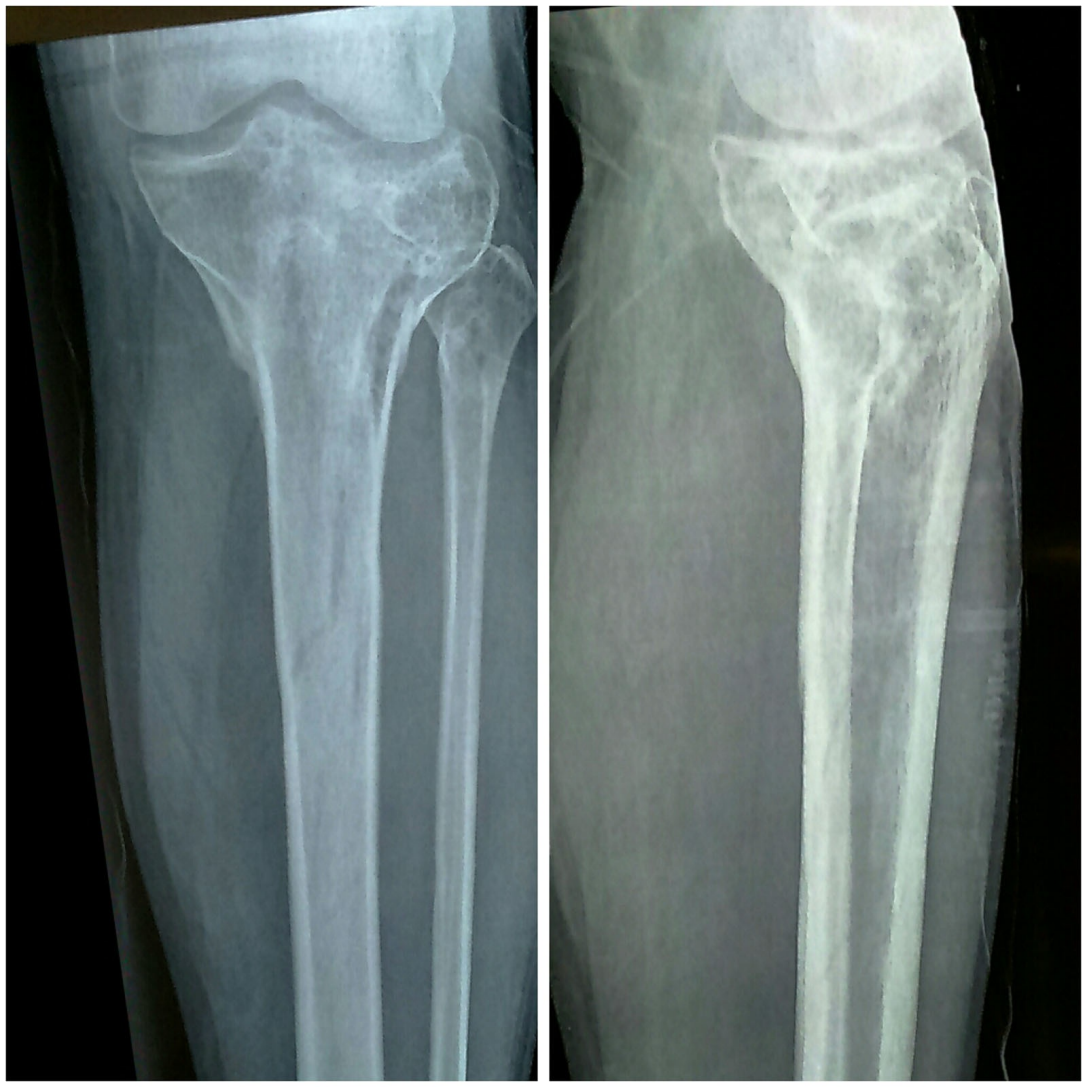
Anteroposterior and lateral X-ray views at eight months of follow-up.

Traction was continued postoperatively. One week after the surgery, knee bending and quadriceps exercises were initiated and exercises were continued on traction for another two weeks. Traction was discontinued and an above knee POP cast was applied for another three weeks following which aggressive knee range-of-motion exercises were started. However, weight bearing was not allowed until after 12 weeks. At eight months of follow-up, an X-ray showed a well-consolidated fracture site (Figure 5).

**Figure 6:**
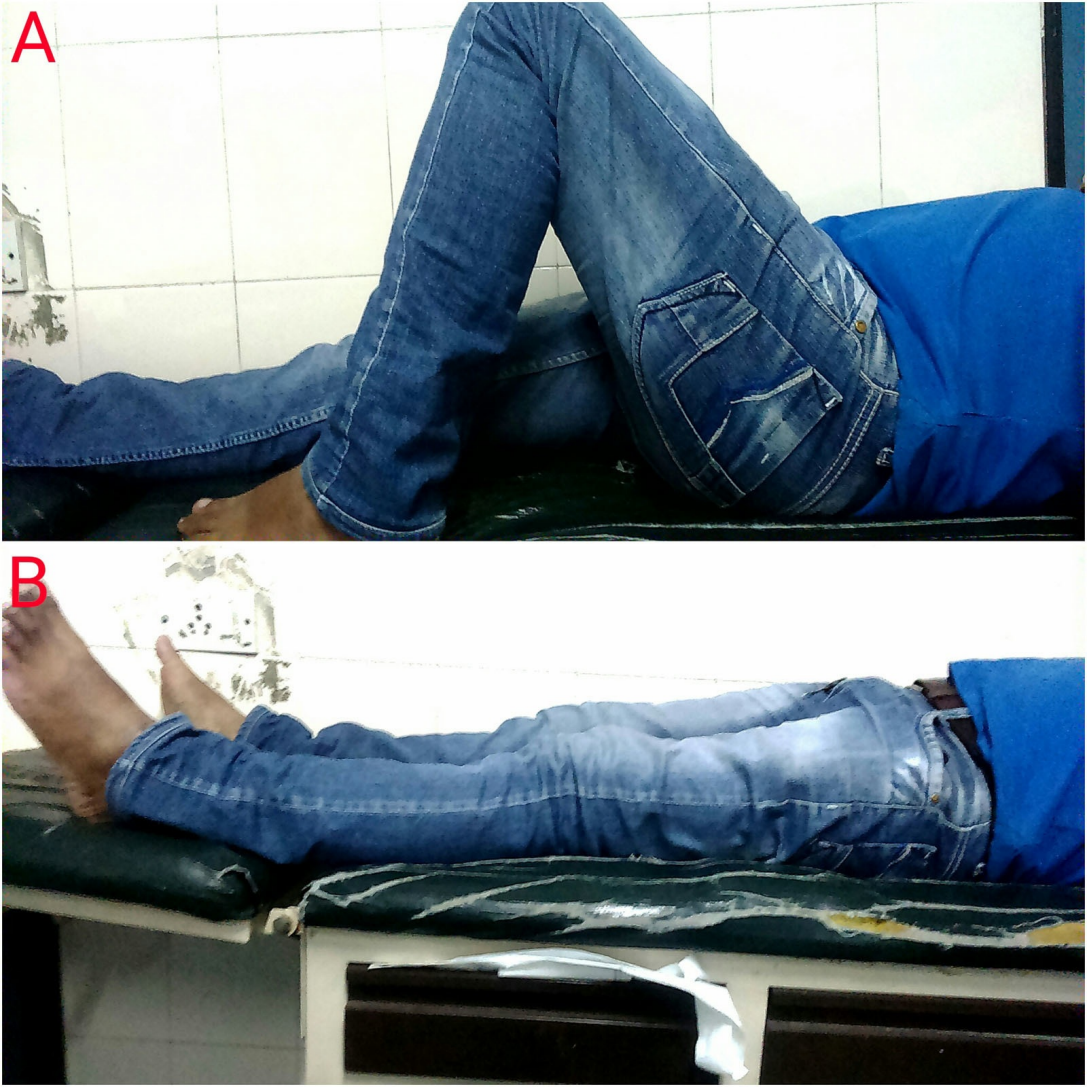
Flexion and extension range of motion at eight months of follow-up.

The patient was pain free and walked unassisted, bearing full weight, with a reasonably good functional range of motion and terminal limitation of squatting (Figure 6). Local examination did not reveal any signs of ligamentous laxity.

## Discussion

Though the treatment of tibial plateau fracture has been under discussion for decades, the ideal treatment of high-energy tibial plateau fractures remains controversial. Most authors prefer ORIF or external fixator for Schatzker type V and VI fractures, providing rigid fixation and early mobilization [1, 8-10].

An uncontrolled diabetes mellitus has been associated with severe wound complications including delayed healing, dehiscence and infection [5]. A surgery involving extensive soft tissue dissection should therefore be undertaken only when the benefits clearly outweigh the risks involved. A risk to benefit ratio of ORIF and conservative management must therefore be considered for individual fracture pattern in such patients.

The advantage of the above method appears that, following a minimally-invasive surgery, the patient was neither immobilized in cast nor bedridden in traction for a duration long enough to cause respective complications of knee stiffness or being bedridden. Because the fracture fragments were held in place by the K-wires, traction could be discontinued once the fracture was gummy. The complications associated with extensive soft tissue damage were also eliminated.

This method cannot be generalized for all type VI Schatzker fractures and is indicated in patients without excessive comminution or severe central depression and with a large peripheral fragment that could be levered to correct the tilt and restore anatomical or near anatomical reduction of the articular surface. Patients with severely depressed fractures and severe comminution should be treated with open reduction and internal fixation, as closed reduction is not feasible. Midway between the two, this method appears to be an updated conservative treatment with minimal surgical manipulation and in which the hospital stay is minimized by applying a cast brace following a short period of traction.

## Conclusions

Minimal intervention with K-wires used appropriately can yield good functional outcomes avoiding the consequences of extensive surgical soft tissue insult, especially in patients with coexistent morbidities like diabetes mellitus and is a valid alternative when ORIF is undesirable due to associated complications. Though the technique appears to be more biological, requiring less surgical time, with a better functional outcome and devoid of major complications, it is restricted to a single case discussed above. Hence, further well-designed, larger randomized trials involving the technique in similar fracture patterns are warranted.
